# Trans-Axonal Signaling in Neural Circuit Wiring

**DOI:** 10.3390/ijms21145170

**Published:** 2020-07-21

**Authors:** Olivia Spead, Fabienne E. Poulain

**Affiliations:** Department of Biological Sciences, University of South Carolina, Columbia, SC 29208, USA; spead@email.sc.edu

**Keywords:** axon–axon communication, growth cone, guidance, fasciculation, adhesion, repulsion, axon sorting, topographic maps

## Abstract

The development of neural circuits is a complex process that relies on the proper navigation of axons through their environment to their appropriate targets. While axon–environment and axon–target interactions have long been known as essential for circuit formation, communication between axons themselves has only more recently emerged as another crucial mechanism. Trans-axonal signaling governs many axonal behaviors, including fasciculation for proper guidance to targets, defasciculation for pathfinding at important choice points, repulsion along and within tracts for pre-target sorting and target selection, repulsion at the target for precise synaptic connectivity, and potentially selective degeneration for circuit refinement. This review outlines the recent advances in identifying the molecular mechanisms of trans-axonal signaling and discusses the role of axon–axon interactions during the different steps of neural circuit formation.

## 1. Introduction 

The formation of neural circuits is a complex developmental process that gives rise to intricate and precise networks essential for brain function [1,2]. Defects in axonal connectivity have been associated with a number of neurological disorders including Autism Spectrum Disorders [3,4], congenital mirror movements [5,6], horizontal gaze palsy with progressive scoliosis [7], and others [8,9,10], making it crucial to better understand the mechanisms governing axon guidance and neural circuit wiring.

During development, axons navigate along precise pathways to reach their final target by responding to attractive or repulsive guidance cues present in their environment. This navigation is ensured by motile structures at their leading ends, the growth cones, which possess numerous receptors at their surface allowing them to respond to the various extra-cellular signals they encounter [11,12]. A panoply of factors are known to provide long-range and/or contact-mediated signals, including the classical guidance cues Ephrins, Slits, Netrins, and Semaphorins [13], adhesion molecules [14,15], neurotrophic and growth factors [16], or morphogens such as Sonic Hedgehog (Shh), Bone morphogenetic proteins (BMPs), and Wnts [17,18]. The activity of these signaling factors has mostly been studied in classical model systems for axon guidance such as the sensory and motor innervation of the limb [19], the midline [20,21], and the retinotectal and olfactory systems [22,23,24,25]. These models have been instrumental in defining the general rules of axon navigation and targeting. For instance, studying the formation of retinotectal maps in the frog led Sperry to formulate his chemoaffinity hypothesis [26], whereby retinal axons with a unique profile of receptors interpret guidance cues distributed in a gradient at their target. While axon–target and axon–extracellular environment interactions have been widely recognized as essential for the formation of circuits, communication between axons themselves has recently emerged as another crucial, yet understudied, mechanism [27,28]. 

The first indication for a role of axon–axon communication, or trans-axonal signaling, in circuit wiring came from early experiments in chick, amphibians, and mice, showing that sensory projections in the limb were disturbed following motor neuron ablation [29,30,31], or that retinal and olfactory axons were still able to order themselves in the absence of their respective target [32,33]. Additional studies in vertebrates and invertebrates have highlighted the importance of neuronal birth timing and age-related axonal elongation in axon–axon communications, with early-born pioneer axons often dictating the trajectory of later-born axons that follow them [34,35]. In the retinotectal system, for instance, pioneer retinal axons guide follower axons out of the eye, at the midline and along the optic tract [36,37]. In addition to the temporal control of axon outgrowth, the temporal regulation of receptor availability at the surface of axons is critical in determining the sensitivity of axons to signals presented by other axons. It also allows axons to change their responsiveness to a cue in a precise spatial and temporal manner [11,38]. While homotypic and heterotypic interactions between axons have been recognized as essential for proper neuronal connectivity for more than four decades, our understanding of the cellular and molecular mechanisms at play has only more recently progressed. In this review, we describe specific examples that highlight the importance of trans-axonal signaling in circuit wiring during development, focusing on the mechanisms underlying selective fasciculation and adhesion, repulsion, and selective defasciculation.

## 2. Axon Fasciculation and Adhesion

Homotypic and heterotypic fasciculation between axons facilitates and coordinates the formation of tracts en route to a common target or between inter-connected brain regions. Early studies demonstrating the importance of pioneer-follower axon interactions in neural circuit formation have suggested a crucial role for adhesion and fasciculation between axons as they navigate towards their target. For example, in the Drosophila olfactory system, ablation of early-born olfactory receptor neurons, whose axons act as pioneers, prevents the targeting of later-born axons to the antennal lobe structure [39]. Similarly, ablation of early-born pioneer neurons in the olfactory [40] or visual [36] systems in zebrafish causes later-born follower axons to misroute on their way to their target and fail to establish proper connections. Interestingly, in the zebrafish forebrain commissure, leading and following commissural axons show a difference in kinetics at the midline: while leading axons pause slightly before speeding back up to cross the midline, followers do not slow down and continue across the midline at a constant speed [41]. Follower axons notably change their kinetics at the midline and adopt a leader behavior upon ablation of leading axons, suggesting a direct interaction between leading and following axons, with follower axons fasciculating and navigating along the pioneering axons. Interestingly, axon fasciculation can not only occur after a growth cone encounters the shaft of a neighboring axon and moves along it, but also through a “zippering” mechanism involving direct interactions between axonal shafts [42]. Overall, interactions between axons are facilitated by cell birth order, expression of adhesion molecules, and conserved signaling pathways that mediate proper fasciculation as axons elongate to their target. 

### 2.1. Homotypic Fasciculation

**Figure 1:**
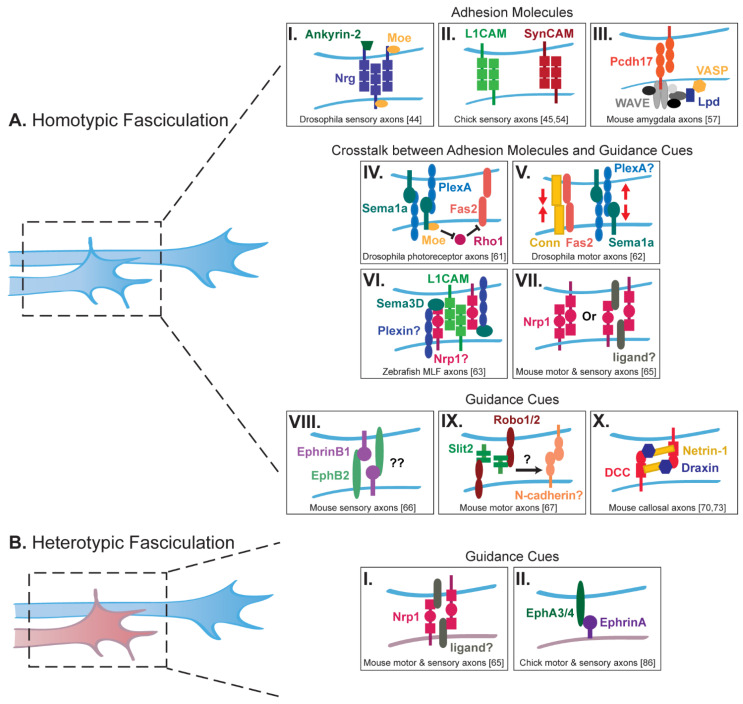
Trans-axonal signaling mediates homotypic (**A**) and heterotypic (**B**) fasciculation. (**AI–III**) Cell adhesion molecules (CAMs) regulate homotypic axon–axon fasciculation. (**AI**) Neuroglian (Nrg) is clustered along the surface of Drosophila sensory axons to mediate homotypic fasciculation [44]. (**AII**) L1CAM and SynCAM both mediate homotypic fasciculation of chick sensory axons [45,54]. (**AIII**) Pcdh17 facilitates trans-axonal homotypic fasciculation in mouse amygdala axons by recruiting the WAVE complex, Lamellipodin (Lpd), and VASP [57]. (**AIV–VII**) Crosstalk between CAMs and classical guidance cues regulate homotypic axon–axon interactions. (**AIV**) Sema1a reverse signaling increases the adhesive function of Fas2 in Drosophila photoreceptor axons [61], while it balances the adhesive functions of Connectin (Conn) and Fas2 in motor axons (**AV**) [62]. (**AVI**) Sema3D signaling likely involving Nrp1 and Plexin co-receptors regulates the expression levels of L1CAM to mediate homotypic fasciculation of zebrafish medial longitudinal fascicle (MLF) axons [63]. (**AVII**) In mouse motor and sensory axons, Nrp1, either on its own or with an unknown ligand, facilitates homotypic axon–axon fasciculation [65]. (**AVIII–X**) Other classical guidance cues also mediate homotypic trans-axonal signaling. (**AVIII**) Ephrin-B1 and EphB2, expressed on mouse sensory axons, may regulate homotypic axon fasciculation by binding in *trans* [66]. (**AIX**) Slit/Robo signaling mediates homotypic fasciculation of motor axons, possibly by regulating the surface levels of N-cadherin [67]. (**AX**) Interactions between Netrin-1, Draxin and DCC facilitate fasciculation of mouse callosal axons [70,73]. (**BI–II**) Classical guidance cues also mediate heterotypic trans-axonal signaling. Nrp1, through an unknown ligand, as well as Ephrin-A/EphA signaling, mediate trans-axonal heterotypic fasciculation of motor and sensory axons [65,86].

Cell adhesion molecules (CAMs) are highly conserved across species and have been found to regulate both homotypic and heterotypic axon–axon interactions in several circuits (Figure 1) [14,15,43]. For example, Neuroglian (Nrg), the Drosophila ortholog of L1-CAMs in vertebrates, is required for the fasciculation between axons projecting to the peduncle of the mushroom bodies [44]. Two intracellular adaptor proteins, Ankyrin-2 and Moesin, interact with Nrg to cluster it along the axonal surface, thereby allowing for stable homophilic Nrg complexes to form between axons as they elongate (Figure 1AI). Similarly, the adhesion molecule L1 facilitates fasciculation between sensory axons during hindlimb development in chick, allowing them to form bundles important for the guidance of later-growing axons [45]. Indeed, injecting an anti-L1 antibody into the chick hindlimb at a timepoint when all motor axons, but only a few sensory axons, have reached the plexus, causes sensory axons to defasciculate and fail to navigate along their proper peripheral nerve [46]. While injecting an antibody against the neural cell adhesion molecule (NCAM) does not alter sensory axon projections, the enzymatic removal of polysialic acid (PSA) from NCAM causes an increase in sensory axon fasciculation, demonstrating that post-translational modifications of CAMs can modulate axon–axon interactions [46]. Similar observations have been made in the neuromuscular and visual systems: in ovo injection of an anti-L1 or an anti-NCAM antibody directly into the iliofibularis muscle in chick leads to the defasciculation of motor axons, while removal of PSA from NCAM increases their fasciculation [47]. Likewise, injecting an anti-NCAM antibody into the developing chick retina causes a disorganization of the optic nerve suggestive of retinal axon defasciculation [48].

Along with L1 and NCAM, early studies in the chick and goldfish retinotectal systems indicate that the adhesion molecule ALCAM (also called BEN, DM-GRASP, SC1, or Neurolin) is also necessary for the proper bundling of retinal axons into fascicles [49,50]. Adding an antiserum against ALCAM to chick retinal explants in culture blocks the elongation of retinal axons on other retinal axons, but not on laminin [49]. Similarly, injecting an anti-ALCAM antibody into the eyes of growing goldfish causes retinal axons to defasciculate, leading to disorganized optic nerves [50]. Interestingly, *ALCAM* mRNA is locally translated in growth cones of retinal axons, and reducing ALCAM local translation in chick retinal axons in culture prevents axons from elongating on ALCAM, but not laminin, coverslips [51]. Retinal axons also show fasciculation defects in *Alcam* knock-out mice [52], indicating that ALCAM’s function in mediating adhesion between axons is conserved across species. 

Other members of the immunoglobulin superfamily of CAMs are also known to regulate axon fasciculation. In the mouse visual system, DSCAM (Down Syndrome Cell Adhesion Molecule) is necessary for the fasciculation of retinal axons along the optic tract [53]. Retinal axons defasciculate in *Dscam* mutants, causing them to stray from their normal path. In the chick peripheral system, the Synaptic cell adhesion molecules SynCAMs were found to regulate axon–axon contacts between afferent fibers as they enter the dorsal root entry zone (DREZ) of the spinal cord [54]. Both overexpression and knockdown of SynCAMs lead to disorganized axon–axon contacts between sensory afferents. In particular, knockdown of SynCAM2 and SynCAM3 leads to the segmentation of axon bundles and mistargeting of axons to the dorsal part of the spinal cord as well as aberrant pathfinding at the DREZ. Finally in the mouse motor system, Contactin-2 (also known as TAG-1) has been detected in the distal segment of motor axons as they elongate into the periphery [55]. Specific inactivation of *TAG-1* in motor neurons causes a thickening of the ventral root of the spinal cord and a defasciculation of motor axons in vitro. 

In addition to the immunoglobulin superfamily of CAMs, members of the cadherin superfamily form another class of CAMs that mediate neural circuit formation through axon–axon interactions. In *C. elegans, fmi-1*, the ortholog of vertebrate Celsr and Drosophila *flamingo,* is needed for adhesion between pioneer and follower axons that form the ventral nerve cord [56]. More recently in the mouse, Protocadherin-17 (Pcdh17) has been shown to be required for growth cone migration at axon–axon contact sites between amygdala axons as they extend to the hypothalamus and ventral striatum [57]. Pcdh17 accumulates at homotypic contacts between cells, and growth cones lacking Pdch17 are no longer able to migrate properly along other axons both in vivo and in vitro. Conversely, ectopic expression of Pcdh17 in axons that do not normally express Pcdh17 causes these axons to mix with axons expressing endogenous Pcdh17. Elegant live imaging and biochemical experiments further showed that Pcdh17 recruits the WAVE complex and the actin-associated proteins Lamellipodin (Lpd) Ena/VASP to axon–axon contact sites, thereby promoting the motility of growth cones as they make contact with other axons of the same tract. 

The function or abundance of adhesion molecules at the axonal surface is tightly regulated by classical guidance cues (Figure 1AIV–VII). In particular, Semaphorins have been identified as major regulators of axon fasciculation. In the Drosophila visual system, transmembrane Sema1a and PlexA are both expressed on the surface of photoreceptor axons that project to the medulla of the optic lobe. While Semaphorins usually act as ligands activating Plexin receptors [58,59], in this system Sema1a reverse signaling mediates axonal adhesion as photoreceptor axons extend through intermediate target zones to the lamina of the optic lobe [60,61]. Interestingly, Sema1a reverse signaling increases the adhesive function of Fascilin 2 (Fas2) at the surface of photoreceptor axons by down-regulating the activity of the small GTPase Rho1 [61]. This is in sharp contrast to its role in the Drosophila motor system, where Sema1a reverse signaling activates Rho1 to promote axon–axon repulsion, thus balancing the adhesive activity of Fas2 and another CAM, Connectin (Conn), for proper motor axon targeting [62]. A similar mechanism whereby Semaphorin signaling regulates the expression of an adhesion molecule has been described in zebrafish [63]. Knockdown of both Sema3D and L1-CAM leads to a loss of adhesion between medial longitudinal fascicle (MLF) axons. Importantly, Sema3D regulates the axonal levels of L1: Sema3D overexpression increases L1 protein levels, whereas Sema3D knockdown decreases them. Which signaling pathway is activated by Sema3D to regulate L1 protein levels remains unclear, but it could involve the Semaphorin-3 receptor Neuropilin-1 (Nrp1). Interestingly, Nrp1 and L1 were found to associate via their extracellular domains [64], and Nrp1 is known to be required for proper homotypic fasciculation of both motor and sensory axons in the developing limb in mice [65]. Altogether, these different studies highlight the control of adhesion molecules by Semaphorin signaling as a key conserved mechanism for regulating homotypic axon fasciculation.

Other classical guidance cues have been shown to mediate homotypic fasciculation, however it remains unknown whether they do so directly or by regulating adhesion molecules (Figure 1AVIII–X). For example, fasciculation in the sensorimotor system of mice is, at least in part, mediated through Ephrin-B/EphB signaling [66]. Ephrin-B1 is present on developing sensory axons as well as in the surrounding limb bud mesenchyme while its receptor, EphB2, is expressed on both sensory and motor axons. Mice lacking Ephrin-B1 exhibit defasciculated sensory and motor axons, supporting a model whereby Ephrin-B1 signaling from the mesenchyme maintains the fasciculation of peripheral projections through a surround-repulsion mechanism. However, defasciculation appears more robust in full knock-out embryos compared to conditional knock-outs lacking Ephrin-B1 from the mesenchyme, suggesting that Ephrin-B1 might also promote the fasciculation of sensory axons via a distinct mechanism involving axon–axon communication [66]. In addition to EphB2, the guidance cue Slit2 and its receptors, Robo1 and Robo2, are expressed by motor neurons in mice as well. Inactivation of any of these three proteins causes motor axons to prematurely defasciculate before reaching their muscle target at the diaphragm [67]. While it remains unclear how Slit2, a secreted factor, promotes motor axon fasciculation, signaling could possibly involve N-cadherin, whose distribution at the surface of placodal cells in chick is increased by Slit/Robo signaling [68]. Finally, another classical guidance pathway that has recently been implicated in mediating axon fasciculation involves the secreted factors Netrin and Draxin and their receptor DCC [69]. Draxin was first identified as a repulsive guidance cue that also promotes homotypic fasciculation between commissural axons in the mouse corpus callosum [70]. A screen for large extracellular protein interactions and immunoprecipitation assays later demonstrated that Draxin interacts with both DCC and Netrin [71,72]. Crystal structures of Draxin/DCC and Draxin/Netrin-1 complexes were recently analyzed and suggest that Draxin tethers Netrin-1 and DCC together to promote axon fasciculation [73].

Along with classical guidance cues, other signaling molecules regulate axon fasciculation in addition to their other better-known functions. Endocannabinoids, for instance, have been identified as guidance cues regulating axon pathfinding and elongation in mouse and xenopus [74], and deletion of the cannabinoid receptor CB_1_R from mouse cortical neurons in vivo causes a defasciculation of their axons [75,76]. A recent study reported that Kinesin-1 regulates the trafficking and sub-cellular localization of CB_1_R in mouse cortical neurons, thereby modulating endocannabinoid signaling and axon fasciculation [76]. 

### 2.2. Heterotypic Fasciculation

The nervous system comprises many axonal tracts formed by different neuron types. Selective heterotypic fasciculation between different axon types coordinates the development of these tracts into neural circuits (Figure 1B). For example, in the developing cerebral cortex, pioneer axons that form the corpus callosum originate from neurons in the cingulate cortex and guide follower axons arising from the neocortex. While cingulate axons are guided toward and across the midline by cues present in the environment, neocortical axons fasciculate with pioneer axons and use them as a guide [77]. Innervation of the habenula is another developmental process that relies on heterotypic axon fasciculation. The habenula includes the lateral habenula (lHb) that projects axons to monoaminergic nuclei, including dopaminergic nuclei, in the ventral tegmental area (VTA). Conversely, the lHb receives reciprocal dopaminergic inputs from the VTA [78,79,80]. Interestingly, the genetic ablation of the habenula in the mouse leads to the loss of dopaminergic projections to the lHB [81]. Furthermore, physically preventing the outgrowth of habenular axons from the lHb also inhibits the elongation of dopaminergic axons to the lHb in mouse brain hemisections, indicating that lHb axons extending to the ventral midbrain sort and guide afferent dopaminergic axons to the lHb [81]. This pre-target reciprocal trans-axonal signaling is mediated by the adhesive molecule Limbic-system-associated protein (LAMP) present at the surface of lHb axons. LAMP likely engages in homophilic interactions to mediate adhesion, as it is also expressed by dopaminergic neurons projecting to the lHb. 

Another example of interactions between reciprocal, afferent, and efferent axons involves thalamocortical and corticothalamic axons that meet in the subpallium to form the internal capsule before projecting to their respective targets [82]. Several studies have suggested that cortical and thalamic axons may rely on each other for proper guidance in the internal capsule. Mutations affecting thalamic axons were shown to alter cortical axons, and conversely [83]. Further studies later confirmed that reciprocal interactions between thalamocortical axons and pioneer cortical axons that are generated by cells in the subplate are required for the guidance of both thalamocortical and corticothalamic projections in the subpallium and within the neocortex. Genetically ablating the thalamus in vivo causes corticothalamic axons to be misguided along a different trajectory towards the cerebral peduncle [84]. The interaction between thalamocortical axons and pioneer cortical axons in the subpallium is required for proper guidance but relies on a tight temporal control of axon elongation [84]. Pioneer cortical axons indeed reach the lateral subpallium before thalamic axons and must halt their elongation and pause until thalamic axons reach the proper location to guide them. This waiting period is triggered by the transient expression of PlexinD1 by pioneer cortical axons that allows them to pause in response to Sema3E secreted by the radial glia. Conversely in the neocortex, cortical efferent axons are required for thalamic axons to cross the pallial-subpallial boundary [85]. 

In the peripheral nervous system, sensory and motor axons also use heterotypic interactions to extend to their appropriate targets (Figure 1B). Along with mediating homotypic fasciculation of motor and sensory axons, Nrp1 regulates heterotypic fasciculation between motor and sensory axons in the developing limb of the mouse [65]. Specific ablation of Nrp1 from sensory axons causes not only sensory but also motor axons to defasciculate. This observation suggests a model whereby Nrp1 acts with a yet unidentified ligand on sensory and motor axons to mediate their fasciculation. Interestingly, Nrp1 and Sema3A are both expressed by motor and sensory neurons, suggesting that motor axons might also defasciculate in response to increased extra-cellular levels of Sema3A that would occur upon Nrp1 ablation from sensory axons. Additional studies in the chick hindlimb have further demonstrated that motor axons extend first and provide a guiding path for the follower sensory axons [30]. It was later found in vertebrates that earlier-projecting motor neurons express EphA3 and EphA4, while sensory axons express Ephrin-A [86]. Inactivation of EphA3 and EphA4 in mouse motor neurons leads to the loss of sensory axon projections. Moreover, the EphA3 ectodomain was shown to promote sensory axon elongation in an Ephrin-A-dependent manner in vitro, suggesting that Ephrin-A reverse signaling mediates interactions between sensory and motor axons. Altogether, these studies highlight the importance of heterotypic axon–axon fasciculation in the establishment of peripheral nerves.

## 3. Trans-Axonal Repulsion 

As important as selective homotypic and heterotypic fasciculation, trans-axonal repulsive interactions mediate the segregation of axons within a tract or the dissociation of axons into distinct tracts forming different circuits. Typically, repulsion between homotypic axons leads to their segregation and sorting en route to or at their target, while heterotypic repulsion generates independent axonal tracts [28]. Repulsion between axonal arbors from same-type neurons also leads to the formation of separate projection fields at the target, thereby ensuring optimal spatial coverage required for efficient connectivity [27,87].

### 3.1. Repulsion and Selective Defasciculation at Choice Points

**Figure 2:**
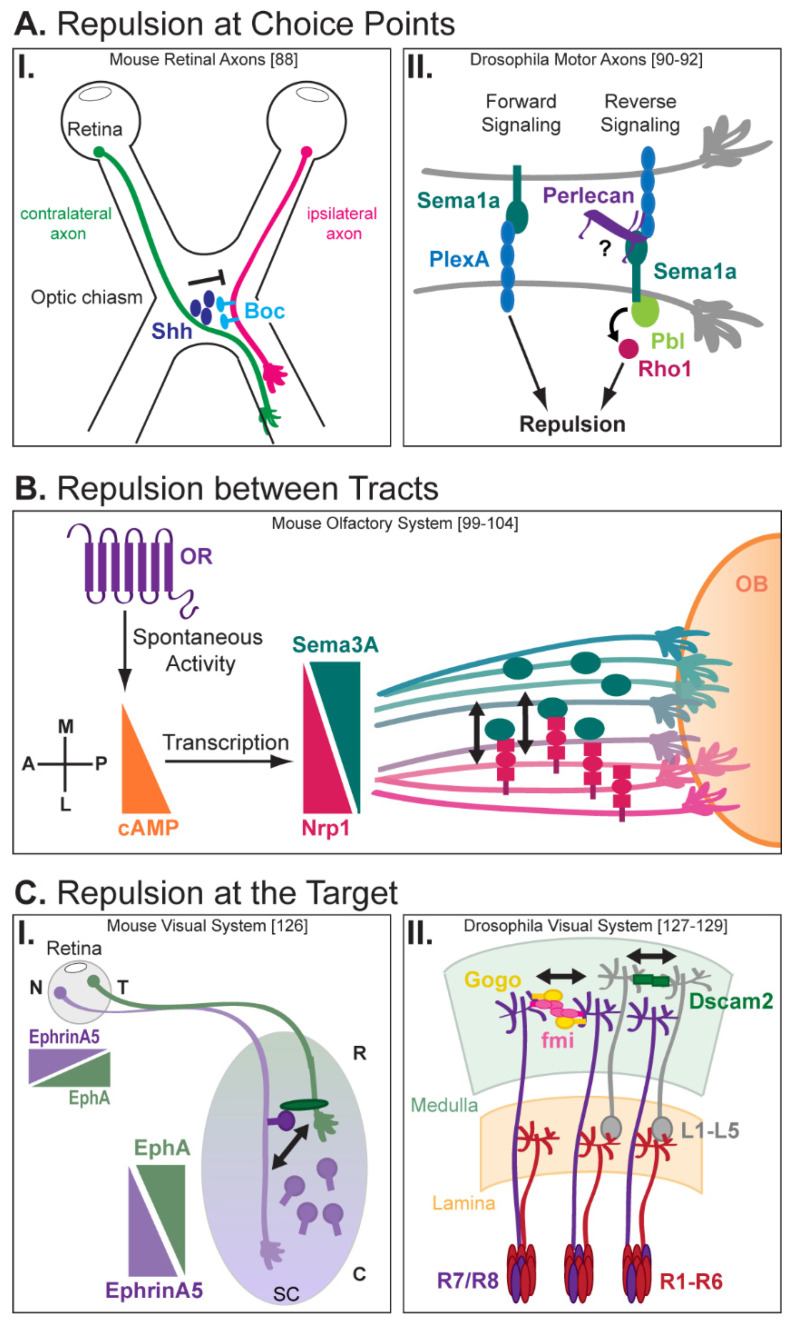
Axon–axon interactions regulate axonal repulsion during neural circuit wiring. (**AI**) In the mouse visual system, contralateral retinal axons arriving early at the optic chiasm secrete Sonic Hedgehog (Shh), which repels later-arriving ipsilateral axons that express the Shh receptor Boc [88]. (**AII**) Both forward and reverse Sema1a signaling regulate motor axon repulsion in Drosophila. Reverse signaling relies on the activation of Rho1 by Pebble (Pbl), and possibly secreted Perlecan [90,91,92]. (**B**) In the mouse, axon–axon repulsion establishes pre-target axon sorting of olfactory sensory axons. ORs produce patterns of spontaneous activity that generate different levels of cyclic adenosine monophosphate (cAMP). cAMP then activates transcription of Nrp1, which is expressed in a complementary manner to its ligand, Sema3A, in the olfactory nerve. Repulsive signaling between Nrp1- and Sema3A-expressing axons sorts axons as they extend to the olfactory bulb (OB) [99,100,101,102,103,104]. (**CI**) Repulsion between nasal and rostral retinal axons at the superior colliculus (SC) contributes to topographic mapping. EphrinA5 is highly expressed by nasal retinal axons while EphA is high on temporal retinal axons. Temporal axons are repelled from the caudal SC by EphrinA5 present in both the environment and at the surface of nasal axons [126]. (**CII**) In the Drosophila visual system, Dscam2 interactions mediate repulsion and proper spacing of L1-L5 axon arbors in the medulla. Spacing of R7/R8 axon arbors is mediated by Flamingo (Fmi) and Golden goal (Gogo) interactions [127,128,129].

As they navigate to their final destination, axons often encounter several successive choice points where intermediate targets such as guidepost cells steer them along the proper path. Interestingly, axons themselves also provide directional information to other axons at choice points (Figure 2A). In the mouse visual system, axons from retinal ganglion cells (RGCs) either cross the midline at the optic chiasm to project contralaterally to the opposite optic tract or do not cross and project ipsilaterally. A recent study demonstrated that contralateral retinal axons that reach the chiasm first transport and secrete Shh at the optic chiasm, thereby repelling later-extending ipsilateral axons that express the receptor Boc (Figure 2AI) [88]. Thus, in this system, trans-axonal repulsive signaling between contralateral and ipsilateral axons provides a spatiotemporal regulation of axon segregation at the optic chiasm that ensures proper subsequent axonal targeting. 

Axon–axon repulsion and selective defasciculation at choice points also allow a subgroup of axons to segregate from the main bundle and navigate to an independent target. Periphery muscle innervation by motor axons, for instance, requires selective axon defasciculation. It has been extensively studied in Drosophila, where motor axons initially exit the central nervous system (CNS) as the intersegmental nerve (ISN) and segmental nerve (SN) before further dividing into five distinct motor branches—the ISN, ISNb, ISNd, SNa, and SNc [89]. Each of these five bundles defasciculates again, so that motor axons innervate individual muscle targets. Sema1a signaling has been shown to regulate the defasciculation of motor axons at specific choice points [90]. Both Sema1a and its receptor PlexA are expressed by motor neurons, and ISN and ISNb axons fail to defasciculate from each other and project to improper targets in *sema1a* and *plexA* mutants. Interestingly, forward and reverse signaling are both required for motor axon defasciculation, with Sema1a reverse signaling being modulated by two counteracting Rho1 GTPase regulators [91]. In particular, the Rho guanine nucleotide exchange factor Pebble (Pbl) acts downstream of Sema1a to promote the defasciculation of ISNb axons at their choice points (Figure 2AII). In absence of Pbl, ISNb motor axons become hyperfasciculated, leading to improper targeting. What mechanism could control the specific activation of Sema1a signaling at choice points? Interestingly, a secreted member of the heparan sulfate proteoglycan (HSPG) family, Perlecan, is found at higher levels in the extracellular matrix (ECM) at defasciculation choice points and appears to be expressed by a subset of motor neurons [92]. Like in *sema1a* and *plexA* mutants, motor axons fail to defasciculate in *perlecan* mutants, and re-expressing *perlecan* in neurons rescues axon fasciculation defects. Whether Perlecan is selectively secreted by motor axons at choice points to regulate Sema1a/PlexA signaling and motor axon defasciculation remains to be established.

Complementary to Semaphorin signaling, the cell adhesion receptors Integrins have also been reported to regulate motor axon defasciculation in Drosophila [93]. Mutants lacking Integrin α1 or Integrin α2 have increased fasciculation of ISNb and SNa axons that causes a lack muscle innervation. Interestingly, DCas, the Drosophila member of the Crk-associated substrate (Cas) family known to act downstream of Integrins, is highly detected in developing motor axons [93]. Like in *Integrin* mutants, ISNb axons fail to defasciculate from the ISN bundle in *DCas* mutants and double *Dcas*/+;*Integrin*/+ heterozygotes, suggesting that Integrin signaling through Dcas is needed for proper motor axon defasciculation. Surprisingly, overexpressing DCas in neurons causes a similar hyperfasciculation defect in an Integrin-dependent manner, suggesting that Integrin/DCas signaling regulates both axon–axon and axon–ECM interactions at choice points. How integrin signaling and other pathways co-regulate axon–axon repulsion to direct defasciculation has yet to be determined.

### 3.2. Axon–Axon Repulsion between and within Tracts

Trans-axonal repulsive signaling between heterotypic tracts allows axons to form distinct bundles and take different trajectories towards their respective targets. In the sensorimotor system, for instance, motor and sensory axon bundles elongate closely to each other in a coordinated manner but remain physically segregated through contact-dependent repulsive interactions as they elongate to the muscle and dermis, respectively [30,45]. Interestingly in mice, EphA3 and EphA4 are present at the surface of motor axons while DRG sensory neurons express high levels of Ephrin-A. Disrupting Ephrin signaling by selectively inactivating EphA3 and EphA4 in motor neurons leads to the intermingling of adjacent motor and sensory axons [94], indicating that Ephrin-A/EphA trans-axonal signaling mediates the repulsion between motor and sensory axons required for their segregation.

Similar trans-axonal repulsive interactions are observed in the developing corpus callosum, where axons from the medial and lateral regions of the cortex project to the contralateral medial and lateral cortical regions, respectively. As they elongate across the midline, medial and lateral cortical axons are organized into separate bundles, with medial axons passing through the dorsal part of the corpus callosum and lateral axons elongating through its ventral part. EphA3 is expressed on lateral-projecting axons and repels medial-projecting axons upon contact, thereby ensuring the segregation of the two axonal tracts [95]. Interestingly, Sema3A-Nrp1 signaling also regulates the topographic ordering of callosal axons [96]. Axons extending from the motor and sensory cortex are spatially segregated into distinct bundles in the corpus callosum. Selectively inactivating Nrp1 or Sema3A in neurons from the motor or sensory cortex, respectively, causes defects in axon segregation, with motor and sensory cortical axons intermixing within the corpus callosum. Disruption of callosal axon organization further leads to subsequent defects in contralateral projections and mapping, highlighting the importance of pre-target topographic ordering of axons for proper brain wiring.

Outside of the corpus callosum, pre-target topographic axon sorting has been observed in many other systems and shown indeed to be an essential step for map formation. For example, thalamocortical axons originating from distinct thalamic nuclei are already ordered in the subpallium before reaching the cortex [97]. Selectively disturbing subpallium development without affecting the thalamus or cortex causes thalamocortical axons to intermix en route to their target and subsequently fail to form functional topographic maps in the cortex [98]. Similarly, pre-target axon sorting is critical for the formation of a functional topographic map in the vertebrate olfactory system [99]. In the mouse olfactory system, about one thousand types of olfactory sensory neurons (OSNs) convey odor information perceived in the olfactory epithelium (OE) to the olfactory bulb (OB). Each OSN expresses a single type of G protein-coupled odorant receptor (OR), and same-type OSNs expressing the same OR extend axons that converge onto common individual target sites called glomeruli in the olfactory bulb [100]. As the organization of glomeruli does not correlate with the position of the OSNs in the OE, convergence of olfactory axons onto specific glomeruli along the antero-posterior axis is ensured by pre-target axon sorting (Figure 2B). Interestingly, ORs themselves have an instructive role in the sorting and targeting of axons that is independent of their odor ligand specificity [101,102]. The spontaneous activity of each OR generates a unique level of cyclic adenosine monophosphate (cAMP) that in turn, initiates signal transduction cascades activating the transcription of specific genes [103,104]. cAMP notably positively regulates the expression of Nrp1, causing OSNs to express variable Nrp1 levels. Interestingly, Nrp1 and Sema3A are expressed in a complementary manner in the olfactory nerve. Axons with high levels of Nrp1 elongate along the outer, lateral part of the bundle, while axons with high levels of Sema3A are confined within its center [99]. Specific inactivation of Nrp1a or Sema3A in OSNs causes axons to intermingle and lose their topographic order within the nerve, indicating that repulsive trans-axonal signaling mediated by Sema3A/Nrp1 determines the relative positioning of olfactory axons. A similar repulsive signaling mediated by Eph-Ephrin has been suggested to regulate the segregation of olfactory receptor cell axons in the moth [105]. 

Pre-target axon sorting has also been extensively studied in the visual system. In vertebrates, retinal axons are preordered along the dorso-ventral axis in the optic tract before reaching the tectum, with dorsal and ventral axons elongating along the ventral and dorsal branches of the tract, respectively [106,107,108,109,110]. As in the olfactory and thalamocortical systems, pre-target ordering of retinal axons is thought to facilitate proper topographic mapping at the target [108]. The signaling mechanism mediating optic tract sorting has not yet been determined, but several studies in zebrafish have highlighted an essential role for heparan sulfate (HS) [111,112,113]. In embryos lacking HS due to mutations in the glycosyltransferases Ext2 and Extl3, pre-target sorting is disrupted, with several dorsal axons misrouting along the dorsal part of the optic tract. Although the mechanism by which HS regulates retinal axon sorting is not well understood, it might involve Nrp1 signaling [114]. Indeed, knockdown of Hermes, an RNA-binding protein expressed in RGCs, leads to partial missorting of dorsal axons through the upregulation of Nrp1 expression, suggesting that Nrp1 levels must be temporally regulated for proper sorting. Interestingly, a micro-RNA, miR-124, indirectly regulates the onset of Nrp1 expression in retinal axons in Xenopus, thereby controlling the sensitivity of retinal growth cones to Sema3A [115]. While Sema3A, Nrp1 and several Plexins are known to be expressed in RGCs, their contribution to retinal axon sorting, and possibly trans-axonal signaling in that system, remains to be tested. Additional studies in Xenopus have highlighted the role of axon–axon interactions in optic tract sorting [116]. In vivo, growth cones of dorsal and ventral axons show a range of behavior upon contact with other axons along the tract, including crossing over the contacted axon, fasciculating with it, or following it at a distance. Further experiments in vitro revealed that homotypic contacts between either dorsal or ventral axons usually lead to axon crossing or fasciculation, while heterotypic contacts are usually followed by axon crossing, stalling or retraction. Interestingly, both homotypic fasciculation and heterotypic repulsion appear to be regulated by the cytoplasmic FMR1-interacting protein 2 (CYFIP2). CYFIP2 translocates to the growth cone periphery upon axonal contact, where it interacts with members of the WAVE regulatory complex (WRC) to regulate actin dynamics. Knockdown of CYFIP2 reduces homotypic fasciculation events and increases axonal stalling and retraction after a growth cone contacts an axon in vitro. In vivo, interaction between CYFIP2 and the WRC is required for the sorting of dorsal axons along the tract, which is disrupted in *cyfip2* zebrafish mutants [117]. While the role of CYFIP2 provides insight into the regulation of heterotypic and homotypic axonal interactions during optic tract sorting, the ligands and receptors upstream of CYFIP2 that would mediate trans-axonal signaling in that system remain unidentified. The importance of axonal birth order and the potential role of pioneer vs. follower axon interactions for the segregation of ventral and dorsal axons have also yet to be addressed.

### 3.3. Axon Repulsion at the Target

Axon–axon interactions continue to dictate the pathway taken by axons as they reach their target and subsequently arborize. In the mouse olfactory system, for instance, olfactory axons are segregated along the dorso-ventral axis and maintain their relative position from their exit from the OE to their entry of the OB. Axons from the dorsomedial zone of the OE project to the dorsal part of the OB first and are followed by axons from the ventrolateral zone that project to the ventral OB [100,118,119]. Interestingly, Sema3F and its receptor Neuropilin-2 (Nrp2) are expressed in a complementary graded manner along the dorso-ventral axis in OSNs. Early-arriving dorsomedial axons exhibit high levels of Sema3F, and later-arriving ventrolateral axons express high levels of Nrp2 [120]. Selective inactivation of Sema3F in OSNs does not affect the sorting of dorsomedial and ventrolateral axons en route to the OB, but it causes Nrp2-expressing axons to mistarget to the dorsal region of the OB upon arrival. The detection of Sema3F protein, but not of *Sema3F* transcript, in the outer nerve layer of the dorsal OB suggests an “indirect” trans-axonal signaling model, whereby pioneer dorsomedial axons produce and deposit Sema3F in the dorsal OB, which in turn repels ventrolateral axons and restricts them to the ventral OB [121]. A similar trans-axonal signaling mechanism between early- and later-arriving axons has been described in the Drosophila olfactory system, where antennal ORN axons expressing high levels of Sema1A reach the peripheral antennal lobe first and repel later-arriving maxillary palp olfactory axons, constraining them to central glomeruli [122].

Trans-axonal signaling also dictates the fine mapping of axons at their final destination. Once axons have elongated and reached their target, they form elaborate axonal arbors within specific territories or termination zones, establishing precise synaptic connections essential for an efficient transfer of information. Several studies have demonstrated that competitive axon–axon interactions facilitate different steps of circuit development, including topographic order and mapping [22]. Repulsive interactions between axons at the target regulate the size and shape of individual axonal arbors, thereby restricting them to precise termination zones. In zebrafish, for instance, interactions between sensory axon arbors limit the size of individual arbors and confine them to restricted territories. In the absence of neighboring neurons, sensory axon arbors continue to grow without restriction [123]. Similarly, retinal axons form larger and more complex arbors at the tectum in the absence of neighboring axons [124,125]. Consequently, disturbing axon–axon interactions independently of the target modifies the formation of precise topographic maps at the target. In the mouse visual system, for instance, trans-axonal signaling between nasal and temporal retinal axons contributes to retinocollicular mapping along the antero-posterior axis (Figure 2CI) [126]. Temporal retinal axons that express high levels of EphA project to the rostral superior colliculus (SC) that expresses Ephrin-As at low levels. Conversely, nasal retinal axons with low levels of EphA project to the caudal SC that expresses high levels of Ephrin-As. Interestingly, the targeting of temporal axons is not affected after selectively inactivating EphrinA5 in the SC, but becomes altered and posteriorly shifted to the caudal SC (that is, the area targeted by nasal axons), upon EphrinA5 ablation in both the SC and the retina. Thus, target-independent trans-axonal signaling prevents temporal and nasal axons from forming overlapping termination zones, thus ensuring proper mapping.

Homotypic axon–axon repulsive interactions finally ensure that axonal arbors from neurons sharing the same function do not overlap with each other and are properly spaced. This “tiling” mechanism allows axons to maximize the coverage of an area while minimizing redundancy of targeting [87]. Interestingly, different types of neurons that innervate a common target tile independently of one another, implicating a specificity of signaling and suggesting an essential role for contact-mediated repulsion. Live imaging studies of trigeminal and spinal cord sensory neurons in zebrafish have indeed confirmed that axons repel each other and limit the size of their arborizations through direct contact-mediated repulsion [123]. Several studies in Drosophila have highlighted the role of adhesion molecules in mediating proper spacing between axonal arbors (Figure 2CII). In the visual system, for instance, the atypical cadherin Flamingo (Fmi) enables proper spacing between R8 photoreceptor axons in the medulla by facilitating competitive interactions between adjacent R8 axonal arbors [127]. When *fmi* is mosaically knocked out in R8 cells, growth cones become irregularly spaced and often overlap, suggesting that Fmi mediates repulsive interactions between R8 cells in a cell-autonomous manner. Interestingly, aggregation assays in vitro and clonal analyses in vivo have recently revealed that Fmi interacts in cis with another transmembrane receptor, Golden goal (Gogo) [128]. Like Fmi, Gogo mediates repulsive axon–axon interactions between R8 axons [129], and *fmi* and *gogo* genetically interact to regulate R8 axon targeting in the medulla [128]. As both Fmi and Gogo colocalize at cell–cell contacts when expressed in cultured cells [128], the formation of Fmi-Gogo complexes might be needed for the proper spacing of R8 axonal arbors. Along with photoreceptor cells, L1-L5 neurons in the lamina also project axons to spatially restricted columns in the medulla, with each column containing only one axon of each neuron type [130]. Homophilic binding between Dscam2, a member of the DSCAM family, mediates repulsion between L1 axonal arbors, thereby restricting them to specific columns. In *dscam2* mutants, L1 axons still target the correct layer of the medulla but are no longer restricted to a single column. 

In addition to mediating the correct spacing of axonal arbors in the Drosophila visual system, atypical cadherins have recently emerged as regulators of axonal tiling in vertebrates [131,132]. Among the 70 different protocadherins (Pcdh) identified in mammals, PcdhαC2 is the only Pcdhα isoform expressed in serotonergic neurons. While serotonergic axon terminals are precisely ordered and evenly spaced in their target fields in the basal ganglia and hippocampus, they appear disorganized, tangled, and clumped together in mice lacking PcdhαC2. This phenotype could also be observed upon specific ablation of PcdhαC2 in serotonergic neurons [120], suggesting that Pcdhαc2 mediates homophilic repulsive interactions to promote tiling between serotonergic axon terminals. Interestingly, the Pcdhα gene cluster has been associated with schizophrenia and autism spectrum disorders, suggesting the possible involvement of defective trans-axonal signaling in the etiology of these neurodevelopmental disorders.

## 4. Concluding Remarks and Future Perspectives

Trans-axonal signaling regulates a striking number of developmental processes that are essential for neural circuit wiring. Both homotypic and heterotypic axon–axon interactions not only mediate axon adhesion and bundling for guidance to the proper target, but also defasciculation for pathfinding at important choice points, repulsion within and between tracts for pre-target sorting and target selection, and repulsion at the target for precise synaptic connectivity. Interestingly, one axonal behavior that is notably lacking from this list is selective axon degeneration. Local axon degeneration refines nervous system connectivity in many species, for instance by remodeling axonal projections during metamorphosis in insects, or by pruning mistargeted axons or axonal branches in vertebrates [133]. In the zebrafish visual system, for example, some dorsal retinal axons initially misroute along the dorsal branch of the optic tract, indicating that pre-target sorting of retinal axons is not precisely established during initial pathfinding. Topographic order is eventually achieved through the selective degeneration of these missorted dorsal axons [103]. The observations that axon–axon interactions participate in the segregation of retinal axons along the tract [106] and that ventral axons elongate along the tract first in zebrafish [134], raise the intriguing possibility that ventral axons might trigger the selective degeneration of missorted dorsal axons. As HS functions non-cell-autonomously to trigger this degeneration [103], testing its role in trans-axonal signaling between ventral and dorsal axons might provide clues about the molecular mechanism involved. 

While many studies have highlighted the importance of axon–axon interactions, the signaling mechanisms and cellular dynamics governing trans-axonal communication are only beginning to emerge in vivo. The recent development of innovative genetic, molecular, and imaging techniques will undoubtedly open new avenues of research by enabling the molecular profiling of single neurons, the selective manipulation of specific axons, and the visualization of axon–axon dynamics at high resolution. Characterizing the molecular and cellular mechanisms by which axons communicate with each other remains a key question to address for better understanding how precise and efficient neural circuits are formed and maintained.

## Abbreviations

ALCAM	Activated leukocyte cell adhesion molecule
BMP	Bone morphogenetic protein
CAM	Cell adhesion molecule
cAMP	Cyclic adenosine monophosphate
Cas	Crk-associated substrate
CNS	Central nervous system
Conn	Connectin
CYFIP2	Cytoplasmic FMR1-interacting protein 2
DREZ	Dorsal root entry zone
DSCAM	Down syndrome cell adhesion molecule
ECM	Extracellular matrix
Fas2	Fascilin 2
Fmi	Flamingo
Gogo	Golden goal
HS	Heparan sulfate
HSPG	Heparan sulfate proteoglycan
ISN	Intersegmental nerve
LAMP	Limbic-system-associated protein
lHB	Lateral habenula
Lpd	Lamellipodin
MLF	Medial longitudinal fascicle
NCAM	Neural cell adhesion molecule
Nrg	Neuroglian
Nrp	Neuropilin
OB	Olfactory bulb
OE	Olfactory epithelium
OSN	Olfactory sensory neuron
Pbl	Pebble
Pcdh	Protocadherin
Plex	Plexin
PSA	Polysialic Acid
RGC	Retinal ganglion cell
SC	Superior colliculus
Sema	Semaphorin
Shh	Sonic hedgehog
SN	Segmental nerve
SynCAM	Synaptic cell adhesion molecule
TAG-1	Transient axonal glycoprotein type-1
VTA	Ventral tegmental area
WRC	WAVE regulatory complex
